# Multi-Sensory Integration Impairment in Patients with Minimal Hepatic Encephalopathy

**DOI:** 10.1038/s41598-017-15113-1

**Published:** 2017-11-02

**Authors:** Kyoungwon Seo, Dae Won Jun, Jae-kwan Kim, Hokyoung Ryu

**Affiliations:** 1 Hanyang University, Department of Industrial Engineering, Seoul, 04763 Republic of Korea; 2 Hanyang University School of Medicine, Department of Internal Medicine, Seoul, 04763 Republic of Korea; 3 Hanyang University, Department of Arts & Technology, Seoul, 04763 Republic of Korea

## Abstract

Paper-and-pencil-based psychometric tests are the gold standard for diagnosis of cognitive dysfunction in liver disease. However, they take time, can be affected by demographic factors, and lack ecological validity. This study explored multi-sensory integration ability to discriminate cognitive dysfunction in cirrhosis. Thirty-two healthy controls and 30 cirrhotic patients were recruited. The sensory integration test presents stimuli from two different modalities (e.g., image/sound) with a short time lag, and subjects judge which stimuli appeared first. Repetitive tests reveal the sensory integration capability. Performance in the sensory integration test, psychometric tests, and functional near-infrared spectroscopy for patients was compared to controls. Sensory integration capability, the perceptual threshold to discriminate the time gap between an image and sound stimulus, was significantly impaired in cirrhotic patients with minimal hepatic encephalopathy (MHE) compared to controls (*p* < 0.01) and non-MHE patients (*p* < 0.01). Sensory integration test showed good correlation with psychometric tests (NCT-A, *r* = 0.383, *p* = 0.002; NCT-B, *r* = 0.450, *p* < 0.01; DST-F, *r* = −0.322, *p* = 0.011; DST- B, *r* = −0.384, *p* = 0.002; ACPT, *r* = −0.467, *p* < 0.01). Psychometric tests were dependent on age and education level, while the sensory integration test was not affected. The sensory integration test, where a cut-off value for the perceptual threshold was 133.3ms, recognized MHE patients at 90% sensitivity and 86.5% specificity.

## Introduction

Impairment of cognition is frequently seen in advanced liver disease. Minimal hepatic encephalopathy (MHE) defined as cognitive dysfunction^1^, reduced quality of life^2^, and driving impairments^3^. MHE increases the risk of progression to overt hepatic encephalopathy and is associated with a poor survival rate^4^. Early interventions for MHE patients can improve their quality of life^5^. Hence, early detection of MHE is clinically important.

There are various diagnostic tools for MHE. A psychometric hepatic encephalopathy score (PHES) and repeatable battery for the assessment of neuropsychological status are mostly widely used paper-and-pencil tests which assess cognitive dysfunction in MHE. Critical flicker frequency^6^, electroencephalogram^7^, and functional near-infrared spectroscopy (fNIRS)^8^ are also used to assess cognitive function in MHE patients. Among them, PHES is the gold standard for MHE diagnosis^9^. International Society for Hepatic Encephalopathy and Nitrogen metabolism recommended measuring multiple cognitive domains including processing speed, working memory, verbal memory, visuospatial ability, visual memory, language, reaction time, and motor functions^9^. Note that PHES is focused on only two cognitive domains which are processing speed and visuospatial ability^2^.

On the other hand, multi-sensory integration is a multi-domain cognitive process which integrates various information from different senses. When different sensory stimuli (e.g., image and sound) arise with a very short time lag, sensory integration perceives them as the same event. If sensory integration failed, meaningful perceptual experience in a real-life environment could not be achieved. Therefore, sensory integration is crucial to participate in activities of daily living^10^. Recently, sensory integration capability gets the limelight in various conditions as sensitive surrogate marker to detect early cognitive impairment. For instance, sensory integration malfunctioning in elderly patients leads to severe cognitive deficits^11^. Autistic patients with impaired sensory integration function also showed they have worse social communication skills^12^. Sensory integration capability can be a powerful measure to assess multiple cognitive domains^13^.

However, there is no study whether capability of sensory integration is impaired in liver cirrhosis and MHE patients. To the best of our knowledge, this is the first study applied “sensory integration” concept for the assessment of cognitive function in cirrhosis. Sensory integration test in MHE can add more multifaceted information and give comprehensive insight to understand pathophysiology of MHE^14^. This study explored sensory integration ability to discriminate cognitive dysfunction in cirrhosis.

## Results

### Basic characteristics of demographic/psychometric results

Basic characteristics of the subjects based on demographic and psychometric results were analyzed by analysis of variance (ANOVA) and post-hoc analysis (Table 1). There were no statistical differences in age and education level among the healthy controls, cirrhotic patients (i.e., cirrhotic patients without MHE), and MHE patients (i.e., cirrhotic patients with MHE). Most psychometric tests, such as number connection test-A (NCT-A), number connection test-B (NCT-B), digit symbol test (DST), digit span test-forward (DST-F), digit span test-backward (DST-B), and auditory continuous performance test (ACPT), showed abnormal findings only in MHE patients, not in healthy controls or cirrhotic patients without MHE.

**Table 1:** Basic characteristics on demographic variables and psychometric test results in healthy controls, cirrhotic patients, and MHE patients.

	Healthy controls	Cirrhotic patients	MHE patients	*P* ^†^	*P* ^‡^	*P* ^§^
**Demographic variables**						
Number (male/female)	32(17/15)	20(13/7)	10(6/4)	—	—	—
Age (years)	55.3 ± 4.9	56.6 ± 6.6	55.0 ± 8.0	0.701	0.886	0.495
Education level (years)	11.9 ± 3.7	11.8 ± 3.6	9.5 ± 3.0	0.165	0.064	0.109
**Perceptual threshold of multi-sensory integration (ms)**	122.8 ± 9.5	126.8 ± 10.1	137.1 ± 2.9	0.000	0.000	0.004
Psychometric tests						
NCT-A (time to completion, s)	33.0 ± 12.9	42.9 ± 20.5	68.7 ± 24.1	0.000	0.000	0.000
NCT-A (number of errors)	0.2 ± 0.4	0.2 ± 0.4	0.1 ± 0.3	0.786	0.681	0.494
NCT-B (time to completion, s)	58.3 ± 26.2	79.5 ± 34.4	174.7 ± 61.7	0.000	0.000	0.000
NCT-B (number of errors)	0.8 ± 1.0	0.6 ± 0.8	2.8 ± 1.9	0.000	0.000	0.000
DST (number of correct answers)	50.6 ± 14.3	45.6 ± 13.2	27.1 ± 10.3	0.000	0.000	0.001
DST-F (number of correct answers)	7.3 ± 1.4	7.1 ± 1.2	5.7 ± 1.3	0.007	0.002	0.008
DST-B (number of correct answers)	4.9 ± 1.5	4.8 ± 1.6	3.4 ± 0.7	0.015	0.004	0.014
ACPT (number of correct answers)	58.1 ± 15.7	63.0 ± 15.9	49.6 ± 20.7	0.122	0.164	0.041
ACPT (number of omission errors)	58.1 ± 15.7	59.2 ± 20.9	49.6 ± 20.7	0.370	0.206	0.182
ACPT (number of missing errors)	50.8 ± 15.5	57.5 ± 15.6	45.7 ± 19.7	0.147	0.388	0.066
ACPT (reaction time, s)	0.6 ± 0.1	0.7 ± 0.1	0.7 ± 0.1	0.001	0.000	0.003

Values are mean ± SD. NCT-A: number connection test-A; NCT-B: number connection test-B; DST: digit symbol test; DST-F: digit span test-forward; DST-B: digit span test-backward; and ACPT: auditory continuous performance test.

^**†**^ANOVA analysis, healthy controls v. cirrhotic patients v. MHE patients.

^**‡**^Post-hoc analysis, healthy controls v. MHE patients.

^**§**^Post-hoc analysis, cirrhotic patients v. MHE patients.

### Correlation between sensory integration test and conventional psychometric tests

The perceptual threshold from sensory integration test, which refers to discriminating a time gap between an image and sound stimulus, showed good correlation with conventional psychometric test results (Table 2). Conventional psychometric tests were highly dependent on subject age (NCT-A, *r* = 0.466, *p* < 0.01; NCT-B, *r* = 0.421, *p* < 0.01; DST, *r* = −0.337, *p* < 0.01; DST-F, *r* = −0.319, *p* < 0.05; and DST-B, *r* = −0.278, *p* < 0.05) and education level (NCT-A, *r* = −0.523, *p* < 0.01; NCT-B, *r* = −0.551, *p* < 0.01; DST, *r* = 0.688, *p* < 0.01; DST-F, *r* = 0.452, *p* < 0.01; DST-B, *r* = 0.351, *p* < 0.01; and ACPT, *r* = 319, *p* < 0.05). Older and less educated subjects had a weaker performance on psychometric tests. In contrast, the derived perceptual threshold from sensory integration test was not influenced by either age (*r* = 0.049, *p* = 0.707) or education level (*r* = −0.021, *p* = 0.869).

**Table 2:** Associations of sensory integration test with conventional psychometric tests

	Threshold of sensory integration	
	*r*	*P*
Demographic factors		
Age (years)	0.049	0.707
Education (years)	−0.021	0.869
Psychometric tests		
NCT-A (time to completion, s)	0.383	0.002
NCT-A (number of errors)	0.104	0.421
NCT-B (time to completion, s)	0.450	0.000
NCT-B (number of errors)	0.333	0.008
DST (number of correct answers)	−0.226	0.077
DST-F (number of correct answers)	−0.322	0.011
DST-B (number of correct answers)	−0.384	0.002
ACPT (number of correct answers)	−0.150	0.244
ACPT (number of omission errors)	−0.101	0.437
ACPT (number of missing errors)	−0.115	0.374
ACPT (reaction time, s)	0.467	0.000

NCT-A: number connection test-A; NCT-B: number connection test-B; DST: digit symbol test; DST-F: digit span test-forward; DST-B: digit span test-backward; and ACPT: auditory continuous performance test.

### Diagnostic performance of the sensory integration test in MHE

**Figure 1:**
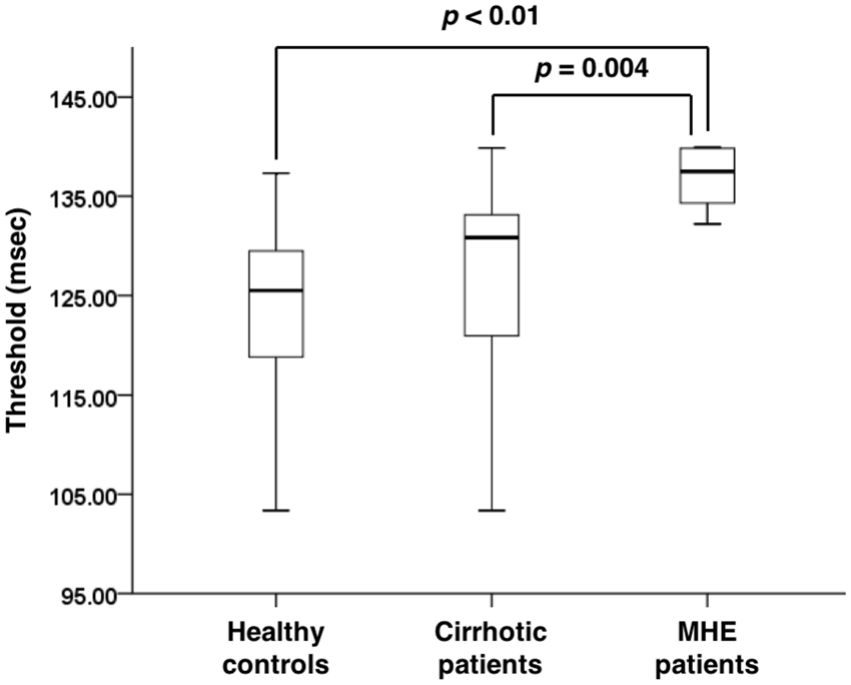
A result of the sensory integration test in each subject group. The box plot shows median (horizontal line within the box), quartiles (upper/bottom line of the box), and min/max values (horizontal line beyond the box). P values from the post-hoc analysis between each subject group are also shown in the figure.

Perceptual threshold was significantly higher in MHE patients than healthy control and cirrhosis without MHE (Fig. 1). Perceptual threshold was 122.8 ± 9.5 ms in healthy controls, 126.8 ± 10.1 ms in cirrhotic patients with normal psychometric results, and 137.1 ± 2.9 ms in MHE patients (F_2,59_ = 9.514, *p* < 0.01). A two-way ANOVA showed no interaction effect between the gender and the subject group on the perceptual threshold (F_2,56_ = 1.015, *p* = 0.369). There was no statistically significant difference in the perceptual threshold between males and females (F_1,56_ = 1.525, *p* = 0.222). The receiver operating characteristic (ROC) curve analysis of the sensory integration test to diagnose MHE patients showed the area under the ROC curve value as 0.937 (95% confidence interval: 0.877~0.996, *p* < 0.01). The sensory integration test with a cut-off value 133.3 ms distinguished between the patients with MHE and the other groups at 90% sensitivity and 86.5% specificity.

### Correlation between sensory integration test and neurophysiological test

Oxygenated hemoglobin (oxyHb) change level was assessed by fNIRS at frontal area (Table 3). Among the fNIRS 16-channels, significant group differences were found in channel 7, channel 10, and channel 13. OxyHb level was significantly lower in MHE patients than both healthy controls and cirrhotic patients.

**Table 3:** Hemodynamic responses in fNIRS channel 7, 10, 13.

	Healthy controls	Cirrhotic patients	MHE patients	*P* ^†^	*P* ^‡^	*P* ^§^
**Hemodynamic responses**						
Channel 7 (OxyHb level)	−0.1 ± 1.3	−0.3 ± 1.4	−1.5 ± 1.0	0.011	0.003	0.021
Channel 10 (OxyHb level)	−0.2 ± 1.5	−0.2 ± 1.8	−1.7 ± 1.1	0.034	0.013	0.022
Channel 13 (OxyHb level)	0.1 ± 1.5	−0.4 ± 1.1	−1.5 ± 1.1	0.008	0.002	0.043

Values are mean ± SD. ^**†**^ANOVA analysis, healthy controls v. cirrhotic patients v. MHE patients. ^**‡**^Post-hoc analysis, healthy controls v. MHE patients. ^**§**^Post-hoc analysis, cirrhotic patients v. MHE patients.

## Discussion

The present study showed that perceptual threshold of multi-sensory integration can discriminate MHE from cirrhosis patients. The sensory integration test was strongly correlated with conventional psychometric tests. This finding reflects that multi-sensory integration is a higher-order cognitive function including multiple cognitive domains such as processing speed, working memory, verbal memory, visual memory, language, reaction time, and motor functions. Hence, the sensory integration test can be a valid measure to assess multiple cognitive domains.

Moreover, sensory integration test had several advantages compared to conventional psychometric tests. First, it lessens the influence from demographic factors. Our results clearly showed that the sensory integration test is not affected by subject’s gender, age, or education level. Contrary to this, conventional psychometric tests are susceptible to demographic factors such as age or education level. These findings corroborate those of other studies suggesting that older people have psychometric decline caused by diminished regional brain volume, myelin integrity, and cortical thickness^15,16^. Association between psychometric results and education level is also well-known^15,17^. Second, the sensory integration test takes less than five minutes to complete. Note that most conventional psychometric tests take more than 15 minutes. Third, the sensory integration test reveals ecologically feasible assessment outcomes which is significantly related with complex real-life situations. The sensory integration capability significantly influence self-management, interaction with others, and complex daily living functions^10^. This is an important finding since the sensory integration test could overcome limitations of conventional clinical tests which lack ecological validity.

In summary, the present study provides preliminary evidence that the sensory integration test is an alternative test to detecting cognitive dysfunction in cirrhotic patients. ROC curve analysis suggested 133.3 ms as the cut-off value of the sensory integration test to discriminate cognitive dysfunction by a good area under the ROC curve value. This finding points to the potential of the sensory integration test as a new screening tool to diagnose MHE patients.

There are some limitations in our study. First, although the sensory integration test discriminated MHE patients from healthy controls, clinical norm data has not been validated for use as a diagnostic tool. Second, it is not yet fully understood how exact MHE-related pathophysiological mechanisms are associated with the impaired sensory integration function. Clinical implications and relations between cognitive dysfunction and sensory integration impairment will be examined in the near future.

Despite these shortcomings, the current study presented the potential of the sensory integration test as a simple yet sensitive tool to diagnose cognitive impairment in cirrhosis irrespective of gender, age, and education level. Until now, most conventional psychometric tests only focused on processing speed and visuospatial ability^2^. This is the first study investigating multi-sensory integration impairment in MHE. The sensory integration test is beneficial not only because the low cost and fast speed of sensory integration test makes it attractive and feasible for wide implementation in a clinical setting, but also because it might help overcome the limitations of many conventional psychometric tests.

## Methods

### Study design

This study was performed as a case-control study. We recruited 32 healthy controls (17 males/15 females) and 30 cirrhotic patients (19 males/11 females) in a single tertiary center. The individual in this manuscript has given written informed consent to publish these case details. A written informed consent form was obtained from each subject after explaining the experimental procedure. This study was approved by the institutional review board of the Hanyang University according to the Declaration of Helsinki (HYUH-2013-08-017-002). The datasets generated during and/or analyzed during the current study are available in the Harvard Dataverse with the DOI: 10.7910/DVN/53W4LE.

### Inclusion criteria

Healthy controls were recruited from a health promotion center. A healthy control was defined after a routine health check-up program. Cirrhotic patients were selected among outpatients from the department of hepatology. Cirrhosis was diagnosed when liver surface nodularity was confirmed by imaging modality or decompensated signs (varix, ascites, or bacterial peritonitis) were observed. Routine laboratory tests, a physical examination, and medical history interview were done by a medical doctor with 17 years of experience.

### Exclusion criteria

Cirrhotic patients who suffered from a disturbance in daily behavior rhythm, such as sleep complaints, daytime somnolence, depression, anxiety, alexithymia, loss of appetite, or gastrointestinal symptoms, were excluded from this study in order to eliminate other confounding factors of cognitive dysfunction. The daily behavior rhythm was assessed using a medical history interview by a medical doctor, where necessary, with additional information from relatives or nursing staffs. None of the subjects abused drugs or drank alcohol heavily within four weeks of starting the study. Other exclusion criteria were a history of psychoactive drug use within four weeks of starting the study, a history of neurological/psychiatric diseases, and a history of brain surgery.

### Psychometric tests

A total of six psychometric tests were administered to the enrolled subjects: (i) NCT-A for assessing both processing speed and motor functions^18^; (ii) NCT-B for language^18^; (iii) DST for visual memory^18^; (iv) DST-F and (v) DST-B for verbal memory and working memory respectively^19^; and (vi) ACPT for reaction time^20^.

Two unbiased medical doctors with 17 and 20 years of experience were employed for this study and they used the psychometric tests to diagnose cirrhotic patients as having MHE (MHE patients) or not having MHE (cirrhotic patients). MHE was diagnosed if at least two psychometric test results exceed 2SDs from those in an age-matched control group^21^. From the 30 cirrhotic patients, 10 patients (six males/four females) were diagnosed as MHE and 20 (13 males/seven females) were diagnosed as cirrhotic patients based on the 2SDs rule.

### Sensory integration test

**Figure 2:**
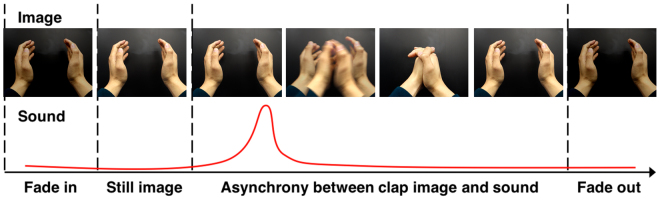
A movie clip of a single clap of two hands which consists of image and sound stimulus. Asynchrony exists between the image and sound stimulus with 10 different time interval conditions. The above figure is one of the sound-first conditions which means a clap sound plays before a clap image.

To evaluate a subjects’ sensory integration function, the sensory integration test was developed based on a previous cognitive neuroscience study^22^. The sensory integration test presents a movie clip of a single clap of two hands which consists of two different sensory modalities such as image and sound (Fig. 2). The stimulus-onset asynchrony (SOA) between the image and sound varied with 10 different time interval conditions such as ±320 ms, ±240 ms, ±160 ms, ±80 ms, and ±40 ms; note that ‘ + ’ means “image-first” and ‘−’ for “sound-first”. Subjects responded to a total of 80 questions where each condition randomly presented eight times.

**Figure 3:**
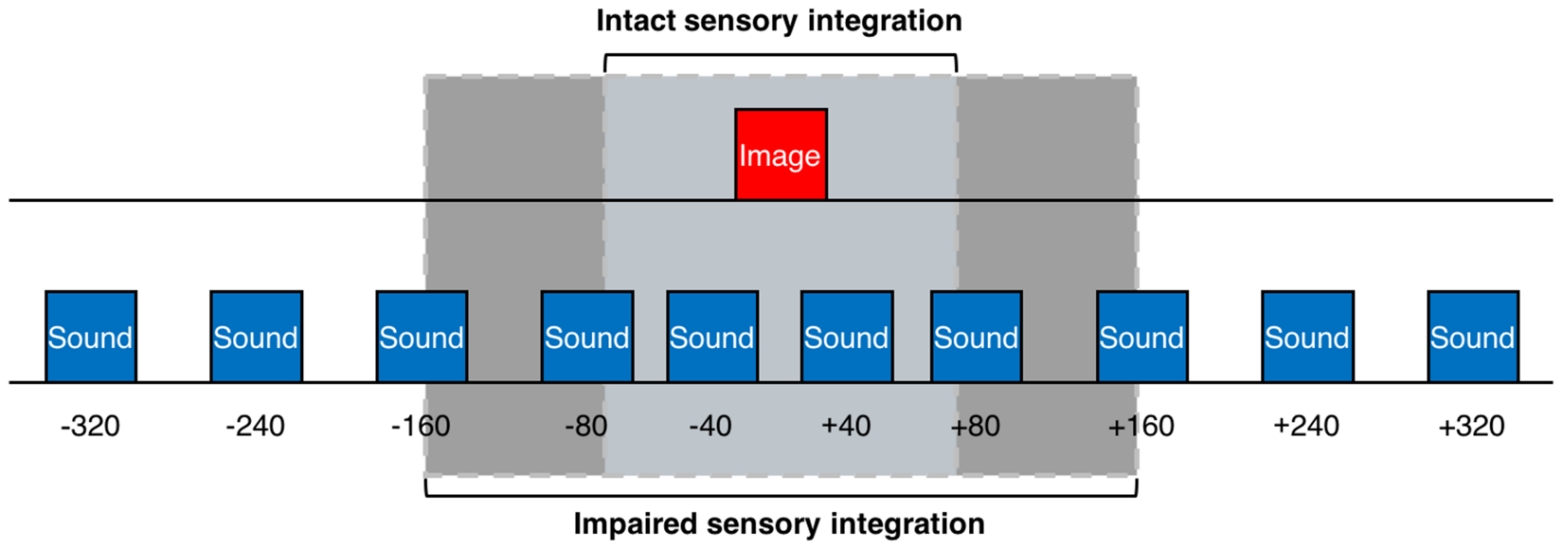
Difference in the perceptual threshold. A smaller perceptual threshold from the sensory integration test indicates that a subject can reliably notice asynchrony between image and sound stimulus based on an intact sensory integration capability. A bigger threshold represents that a subject cannot distinguish asynchrony due to an impaired capability.

In each question, the subject judges whether the image or the sound stimulus appeared first in the movie clip. The responses were plotted in an S-shaped logistic regression curve as the “image-first” response proportion of the SOA. As a result of the sensory integration test, we defined the perceptual threshold as the smallest time lag that a subject can reliably notice between asynchronous image and sound stimulus. The perceptual threshold was calculated from half of the estimated SOA values between the 25% and 75% marks on the curve. Note that the perceptual threshold means a just-noticeable difference. A smaller perceptual threshold indicates an intact sensory integration capability, whereas a bigger threshold indicates an impaired capability (Fig. 3).

### Functional near-infrared spectroscopy

The fNIRS measures the prefrontal hemodynamic responses during the sensory integration test in order to examine the underlying neurophysiological mechanisms in MHE^14^. It is a non-intrusive and on-the-spot functional neuroimaging technique that allows detection of prefrontal brain activity by using near-infrared light. Many studies have used fNIRS due to its convenience and portability^23^.

The 16-channel Spectratech OEG-16 device was used in this study. Each subject’s hemodynamic changes in the prefrontal area were recorded using a sampling rate of 650 ms. The recorded data were converted to oxyHb and deoxygenated hemoglobin (deoxyHb) change level by applying a modified Beer-Lambert law^24^. A pre-process method (e.g., zero-phase filters with a cut-off frequency of 0.01–0.09 Hz) was employed via MATLAB to remove physiological noise such as heartbeat, breathing, and eye blinking^25^. Decreased oxyHb activation is related to the onset of disease symptoms^26^. Therefore, oxyHb change level could be a potential measure of MHE. The 10 s baseline measurement for fNIRS was conducted before the sensory integration test. The oxyHb change level was calculated by the formula (M_n_ − M_bs_)/SD_bs_^27^. M_n_ and M_bs_ denote the mean of observed oxyHb level during the sensory integration test and the first 10 s baseline, respectively. SD_bs_ is the standard deviation of the first 10 s baseline.

### Procedure

The experimental tests were conducted in a quiet room with normal lighting. All subjects performed a training session for sensory integration test with immediate error feedback. The subjects continued the training session until six consecutive correct answers were given. After a training session, subjects continued the sensory integration test without error feedback. Note that subjects wore fNIRS to assess oxyHb change level.

### Statistical analysis

Well-known statistical methods were applied to examine the feasibility and the validity of the sensory integration test as a diagnostic tool for MHE by using IBM SPSS Statistics 21. A one-way ANOVA was applied to show the feasibility of the sensory integration test and fNIRS. A two-way ANOVA was conducted to examine the effect of the gender and the subject group on the sensory integration test. Correlation analysis between conventional psychometric tests and the sensory integration test was employed for validity analysis. A receiver operating characteristic (ROC) curve was also plotted to examine the validity of the sensory integration test.

## References

[1] Bajaj JS, Wade JB, Sanyal AJ (2009). Spectrum of neurocognitive impairment in cirrhosis: Implications for the assessment of hepatic encephalopathy. Hepatology.

[2] Bajaj JS (2008). Minimal hepatic encephalopathy matters in daily life. World J Gastroenterol.

[3] Bajaj JS (2009). Minimal hepatic encephalopathy is associated with motor vehicle crashes: the reality beyond the driving test. Hepatology.

[4] Romero-Gomez M, Boza F, Garcia-Valdecasas MS, Garcia E, Aguilar-Reina J (2001). Subclinical hepatic encephalopathy predicts the development of overt hepatic encephalopathy. Am J Gastroenterol.

[5] Shukla S, Shukla A, Mehboob S, Guha S (2011). Meta-analysis: the effects of gut flora modulation using prebiotics, probiotics and synbiotics on minimal hepatic encephalopathy. Aliment Pharmacol Ther.

[6] Romero-Gomez M (2007). Value of the critical flicker frequency in patients with minimal hepatic encephalopathy. Hepatology.

[7] Amodio P (2008). Detection of minimal hepatic encephalopathy: normalization and optimization of the Psychometric Hepatic Encephalopathy Score. A neuropsychological and quantified EEG study. J Hepatol.

[8] Nakanishi H (2014). Impaired brain activity in cirrhotic patients with minimal hepatic encephalopathy: Evaluation by near-infrared spectroscopy. Hepatol Res.

[9] Randolph C (2009). Neuropsychological assessment of hepatic encephalopathy: ISHEN practice guidelines. Liver Int.

[10] Welch RB, Warren DH (1980). Immediate perceptual response to intersensory discrepancy. Psychol Bull.

[11] Chan JS (2015). Expanded temporal binding windows in people with mild cognitive impairment. Curr Alzheimer Res.

[12] de Boer-Schellekens L, Eussen M, Vroomen J (2013). Diminished sensitivity of audiovisual temporal order in autism spectrum disorder. Front Integr Neurosci.

[13] Pasqualotto A, Dumitru ML, Myachykov A (2015). Editorial: Multisensory Integration: Brain, Body, andWorld. Front Psychol..

[14] Schnitzler A, Gross J (2005). Normal and pathological oscillatory communication in the brain. Nat Rev Neurosci.

[15] Kircheis G, Fleig WE, Gortelmeyer R, Grafe S, Haussinger D (2007). Assessment of low-grade hepatic encephalopathy: a critical analysis. J Hepatol.

[16] Salthouse TA (2009). When does age-related cognitive decline begin?. Neurobiol Aging.

[17] Stern Y (2012). Cognitive reserve in ageing and Alzheimer’s disease. Lancet Neurol.

[18] Weissenborn K (2008). PHES: one label, different goods?!. J Hepatol.

[19] McCrea M, Cordoba J, Vessey G, Blei AT, Randolph C (1996). Neuropsychological characterization and detection of subclinical hepatic encephalopathy. Arch Neurol.

[20] Lockwood AH (2002). Positron emission tomography in the study of hepatic encephalopathy. Metab Brain Dis.

[21] Weissenborn K, Ennen JC, Schomerus H, Ruckert N, Hecker H (2001). Neuropsychological characterization of hepatic encephalopathy. J Hepatol.

[22] Stekelenburg JJ, Vroomen J (2007). Neural correlates of multisensory integration of ecologically valid audiovisual events. J Cogn Neurosci.

[23] Cui X, Bray S, Bryant DM, Glover GH, Reiss AL (2011). A quantitative comparison of NIRS and fMRI across multiple cognitive tasks. Neuroimage.

[24] Baker WB (2014). Modified Beer-Lambert law for blood flow. Biomed Opt Express.

[25] Bauernfeind G, Scherer R, Pfurtscheller G, Neuper C (2011). Single-trial classification of antagonistic oxyhemoglobin responses during mental arithmetic. Med Biol Eng Comput.

[26] Abe K (2016). Reduced frontal activation during verbal fluency task in chronic hepatitis C patients with interferon-based therapy as measured by near-infrared spectroscopy. Hepatol Res.

[27] Ogawa Y, Kotani K, Jimbo Y (2014). Relationship between working memory performance and neural activation measured using near-infrared spectroscopy. Brain Behav.

